# Quality of life analyses in patients with multiple myeloma: results from the Selinexor (KPT-330) Treatment of Refractory Myeloma (STORM) phase 2b study

**DOI:** 10.1186/s12885-021-08453-9

**Published:** 2021-09-06

**Authors:** Gabriel Tremblay, Patrick Daniele, Janis Breeze, Lingling Li, Jatin Shah, Sharon Shacham, Michael Kauffman, Monika Engelhardt, Ajaj Chari, Ajay Nooka, Dan Vogl, Maria Gavriatopoulou, Meletios-Athanasios Dimopoulos, Paul Richardson, Noa Biran, David Siegel, Philip Vlummens, Chantal Doyen, Thierry Facon, Mohamad Mohty, Nathalie Meuleman, Moshe Levy, Luciano Costa, James E. Hoffman, Michel Delforge, David Kaminetzky, Katja Weisel, Marc Raab, David Dingli, Sascha Tuchman, Frenzel Laurent, Ravi Vij, Gary Schiller, Philippe Moreau, Joshua Richter, Martin Schreder, Klaus Podar, Terri Parker, Robert Frank Cornell, Karlin Lionel, Sylvain Choquet, Jagannath Sundar

**Affiliations:** 1 Purple Squirrel Economics, 1600 Notre Dame W, Suite 201, Montreal, QC H3J 1M1 Canada; 2 Karyopharm Therapeutics Inc., Newton, USA; 3 University of Freiburg, Freiburg im Breisgau, Germany; 4 Icahn School of Medicine at Mount Sinai, New York, USA; 5 Winship Cancer Institute, Emory University, Atlanta, USA; 6 Abramson Cancer Center, Perelman School of Medicine, University of Pennsylvania, Philadelphia, USA; 7 Oncology Department, Alexandra Hospital, Athens, Greece; 8 School of Medicine, National and Kapodistrian University of Athens, Athens, Greece; 9 Harvard Cancer Center, Boston, USA; 10 Hackensack Meridian Health Hackensack University Medical Center, Hackensack, USA; 11 University Hospital Ghent, Ghent, Belgium; 12 Université catholique de Louvain, Ottignies-Louvain-la-Neuve, Belgium; 13 University Hospital, Lille, France; 14 Hopital Saint-Antoine, Paris, France; 15 Institut Jules Bordet, Brussels, Belgium; 16 Baylor University Medical Center, Dallas, USA; 17 University of Alabama at Birmingham, Birmingham, USA; 18 Sylvester Cancer Center, University of Miami, Miami, USA; 19 University of Leuven, Leuven, Belgium; 20 New York University Langone Medical Center, New York, USA; 21 University Medical Center Hamburg-Eppendorf, Hamburg, Germany; 22 University of Heidelberg, Heidelberg, Germany; 23 Mayo Clinic, Rochester, USA; 24 Lineberger Comprehensive Cancer Center at University of North Carolina-Chapel Hill, Chapel Hill, USA; 25 Hôpital Necker, Paris, France; 26 Washington University School of Medicine, St. Louis, USA; 27 David Geffen School of Medicine at University of California, Los Angeles, USA; 28 University of Nantes, Nantes, France; 29 Tisch Cancer Institute, Icahn School of Medicine at Mount Sinai, New York, USA; 30 University Hospital Würzburg, Würzburg, Germany; 31 University Hospital Krems, Karl Landsteiner University of Health Sciences, Krems an der Donau, Austria; 32 Yale School of Medicine, New Haven, USA; 33 Vanderbilt University Medical Center, Nashville, USA; 34 Centre Hospitalier Lyon Sud, Saint-Genis-Laval, France; 35 La Pitié Salpêtrière Hospital, Paris, France

**Keywords:** Patient reported outcomes, Health-related quality of life, FACT-MM, Multiple myeloma, Selinexor

## Abstract

**Background:**

Selinexor is an oral, selective nuclear export inhibitor. STORM was a phase 2b, single-arm, open-label, multicenter trial of selinexor with low dose dexamethasone in patients with penta-exposed relapsed/refractory multiple myeloma (RRMM) that met its primary endpoint, with overall response of 26% (95% confidence interval [CI], 19 to 35%). Health-related quality of life (HRQoL) was a secondary endpoint measured using the Functional Assessment of Cancer Therapy – Multiple Myeloma (FACT-MM). This study examines impact of selinexor treatment on HRQoL of patients treated in STORM and reports two approaches to calculate minimal clinically important differences for the FACT-MM.

**Methods:**

FACT-MM data were collected at baseline, on day 1 of each 4-week treatment cycle, and at end of treatment (EOT). Changes from baseline were analyzed for the FACT-MM total score, FACT-trial outcome index (TOI), FACT-General (FACT-G), and the MM-specific domain using mixed-effects regression models. Two approaches for evaluating minimal clinically important differences were explored: the first defined as 10% of the instrument range, and the second based on estimated mean baseline differences between Eastern Cooperative Oncology Group performance status (ECOG PS) scores. Post-hoc difference analysis compared change in scores from baseline to EOT for treatment responders and non-responders.

**Results:**

Eighty patients were included in the analysis; the mean number of prior therapies was 7.9 (standard deviation [SD] 3.1), and mean duration of myeloma was 7.6 years (SD 3.4). Each exploratory minimal clinically important difference threshold yielded consistent results whereby most patients did not experience HRQoL decline during the first six cycles of treatment (range: 53.9 to 75.7% for the first approach; range: 52.6 to 72.9% for the second). Treatment responders experienced less decline in HRQoL from baseline to EOT than non-responders, which was significant for the FACT-G, but not for other scores.

**Conclusion:**

The majority of patients did not experience decline in HRQoL based on minimal clinically important differences during early cycles of treatment with selinexor and dexamethasone in the STORM trial. An anchor-based approach utilizing patient-level data (ECOG PS score) to define minimal clinically important differences for the FACT-MM gave consistent results with a distribution-based approach.

**Trial registration:**

This trial was registered on ClinicalTrials.gov under the trial-ID NCT02336815 on January 8, 2015.

**Supplementary Information:**

The online version contains supplementary material available at 10.1186/s12885-021-08453-9.

## Background

Multiple myeloma (MM) is the second most common form of hematologic cancer in the United States (US), with an estimated 32,110 new cases in 2019 [1]. MM is characterized by the abnormal proliferation of clonal plasma cells in the bone marrow, alterations in the bone marrow microenvironment, and the production of monoclonal protein and other bioactive molecules by malignant cells [2]. Patients with MM experience a burden of symptoms due to clinical manifestations associated with end organ damage, including hypercalcemia, renal insufficiency, renal failure, anemia, immune dysfunction, and bone destruction [3]. Current treatment modalities for MM include proteasome inhibitors, immunomodulatory agents, and monoclonal antibodies, which are often used in doublet or triplet drug regimens, as well as chemotherapy, bone marrow transplant, and radiation therapy [4, 5].

According to the US Surveillance, Epidemiology, and End Results (SEER) database, the 5-year survival rate for patients diagnosed with MM from 2010 to 2016 was estimated to be 53.9% [1]. At present, MM remains generally incurable, and almost all patients relapse and develop refractory disease [6, 7]. With each relapse, patients face worsening clinical outcomes due to declining efficacy of treatment regimens, shorter duration of response, and increased refractoriness to therapeutic agents [6, 8–10].

The refractory nature of MM and severity of symptoms impact quality of life (QoL) and limit availability of treatment options for patients [11–13]. Previous studies have provided evidence of poor QoL among patients with relapsed/refractory MM (RRMM), who face a significant burden of disease and cumulative impacts of prior treatments and treatment-associated adverse events [12]. As patients progress through multiple lines of therapy and exhaust available treatment options with lessening clinical benefit, they may decide between experimental therapy, retreatment strategies, and symptomatic care [14].

Selinexor is a first-in-class selective oral nuclear transport inhibitor that has been approved by the US Food and Drug Administration in combination with low-dose dexamethasone for the treatment of adults with RRMM who have received at least four prior therapies and whose disease is refractory to at least two proteasome inhibitors, two immunomodulatory agents, and an anti-CD38 monoclonal antibody [15]. Efficacy and safety of selinexor in RRMM were demonstrated in the STORM (Selinexor Treatment of Refractory Myeloma) phase 2b trial (NCT02336815; *N* = 122; *n* = 83 with penta-refractory myeloma i.e., refractory to bortezomib, carfilzomib, lenalidomide, pomalidomide, and daratumumab [16]. Results of STORM have been previously published elsewhere [16]. Briefly, the primary endpoint was overall response, defined as a partial response or better, and was observed in 26% of patients (95% CI, 19 to 35%). Among all responders, the median duration of response was 4.4 months. In the modified intent-to-treat (mITT) population, the median overall survival was 8.6 months. The most common adverse events were thrombocytopenia, nausea, fatigue, anemia, decreased appetite, and decreased weight, which were managed with supportive care and dose modifications. Patient-reported outcome (PRO) data were collected using the Functional Assessment of Cancer Therapy – Multiple Myeloma (FACT-MM) instrument at study screening, at each cycle, and at end of treatment. This analysis provides an assessment of patient-reported QoL with selinexor and low dose dexamethasone in the STORM trial. In addition, it aims to evaluate the proportion of patients with minimal clinically meaningful change in QoL from baseline.

## Methods

### Study design and quality of life assessment

The patient eligibility criteria and study design of STORM have been previously described [16]. Briefly, STORM was a phase 2b, multicenter, open-label study of twice-weekly oral selinexor (80 mg) in combination with dexamethasone (20 mg) in patients with progressive MM [16]. The mITT population included 122 patients (median age 65.2 years), of whom 83 (68%) had penta-refractory MM. Patient-reported QoL was a secondary endpoint and was assessed at study screening and on day 1 of each 4-week treatment cycle beginning at cycle 2, and at end of treatment with the FACT-MM. The FACT-MM is a disease-specific instrument and has been previously applied in the assessment of health-related QoL (HRQoL) among patients with RRMM in investigational studies [17–20]. FACT-MM combines the General version of the FACT (FACT-G; 27 items) with an MM-specific subscale (MM domain; 14 items). The MM domain addresses symptomatic burden and disease-specific well-being [19]. The total FACT-MM score is obtained by adding individual subscale scores for physical well-being (7 items), social/family well-being (7 items), emotional well-being (6 items), and functional well-being domains (7 items) of the FACT-G and the MM domain [19]. The FACT-MM Trial Outcomes Index (TOI) is comprised of the physical and functional subscales and the MM domain [19].

### Statistical analysis

The analysis methods have been previously presented in brief in a conference abstract by Breeze et al. [21]. The QoL analysis dataset consisted of 80 patients in the mITT population with FACT-MM data at baseline and at least one follow-up cycle or end of treatment. Baseline characteristics, including demographic information (e.g., age, gender, race) and clinical variables were summarized using means and standard deviations for continuous variables and counts and proportions for categorical variables. Race was collapsed as white, black, or other for inclusion in the analysis. Patients with missing demographic or clinical variable data were omitted from the HRQoL analyses. Completeness was defined according to the FACT-MM Scoring Guidelines (Version 4), which allows subscales to be calculated if > 50% of items are present, and total scores if > 80% of items are present.

For each follow-up cycle, the magnitude of change from baseline was evaluated using mixed-effects regression models, allowing for random slope and intercept terms for repeated measures for the FACT-MM total score, FACT-G, FACT-MM TOI, and the MM domain. This type of regression model assumes data are missing at random (MAR).

Multivariable adjusted models were constructed which considered baseline scores and baseline characteristics including demographic data (age, gender, and race), Eastern Cooperative Oncology Group (ECOG PS) score (categorized as 0 or 1 to 2), and years since diagnosis as prognostic variables. During selection of variables for model inclusion, each model was evaluated for robustness using model fit parameters including Akaike’s Information Criterion (AIC), Bayesian Information Criterion (BIC), and the model chi-square statistic [22, 23]. Final adjusted models which include baseline scores and specified baseline characteristics that improved fit are reported.

The minimal clinically important difference represents the smallest meaningful improvement in the score of a PRO domain, interpreted as a minimum level of perceived benefit by patients, and has been generally utilized in the translation of HRQoL outcomes to clinical practice and treatment choice [24, 25]. To our knowledge, no minimal clinically important difference thresholds have been reported for the FACT-MM. In the current analysis, clinically meaningful changes were evaluated by examination of minimal clinically important difference using two anchor-based approaches. In the first approach, minimal clinically important difference was defined as 10% of the instrument range, a threshold that has been associated with meaningful HRQoL change in patients using cancer-specific instruments such as the FACT-G [26]. An exploratory approach was developed for this analysis where HRQoL was ‘anchored’ to differences in ECOG PS scores, which is a measure used by clinicians to assess and describe the clinical status and prognosis of patients and to guide treatment. Previous anchor-based analyses have used clinical characteristics such as ECOG PS or laboratory findings such as hemoglobin levels to derive MCIDs for disease-specific FACT subscales [27, 28]. In these analyses, adjacent categories in selected characteristics were presumed to represent clinically distinguishable groups within the HRQoL dataset. Following these approaches, the current analysis used patient-level data to group patients into categories based on physician-assessed baseline ECOG PS. Due to the low number of patients included in the analysis, ECOG PS 1 and 2 categories were grouped together. The minimal clinically important difference was thus defined as the difference in mean baseline scores between patients with ECOG PS of 0 compared with those with ECOG PS of 1 to 2, adjusted for significant baseline characteristics (race, age) to account for confounding arising from the non-randomized nature of the ECOG groupings.

For either approach, patients with a minimal clinically important difference improvement were considered as having HRQoL improvement [24]. Patients with less than the minimal clinically important difference change were considered stable. Patients with a minimal clinically important difference decrease were considered as having HRQoL decline [29].

In addition, post-hoc testing was carried out to examine HRQoL trends between treatment responders and non-responders. Responders were defined as patients with overall response (partial response or better). Since these subgroups were not randomized, a difference analysis (a quasi-experimental approach) was used to statistically compare the differences in HRQoL scores from baseline to end of treatment for treatment responders and non-responders. Estimated mean differences between responders and non-responders were directly derived from the mixed effects model.

## Results

**Fig. 1:**
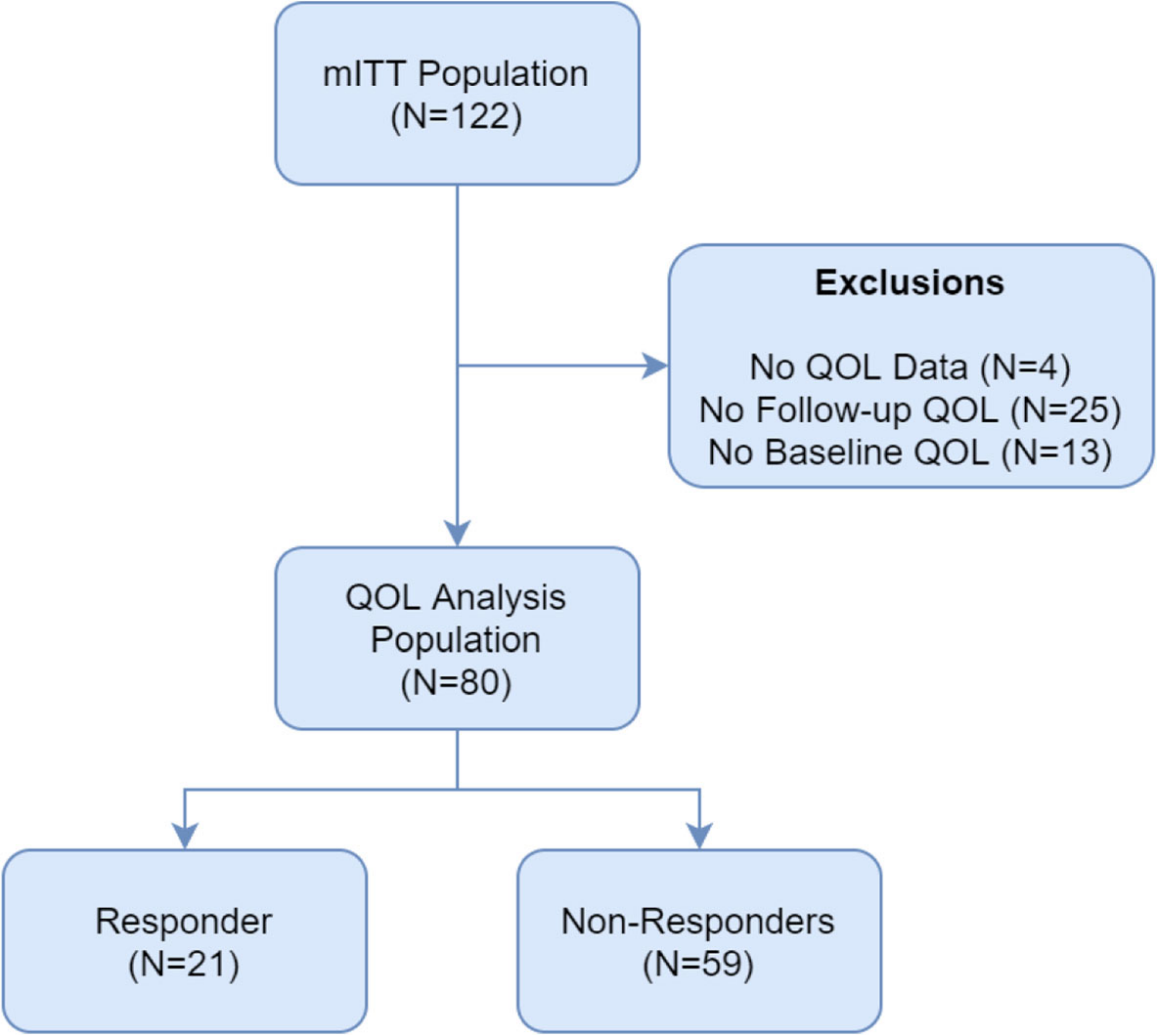
Study flowchart. mITT: modified intent-to-treat; QoL = quality of life

The key results of the analysis were previously reported in a conference abstract by Breeze et al. [21]. Of 122 patients in the STORM mITT population, 80 (66%) completed the FACT-MM at baseline and at one or more follow-up cycle or at end of treatment (Fig. 1). In the QoL analysis population, 21 patients experienced partial response or better and were considered as treatment responders (26%; *n* = 21/80). Baseline characteristics and clinical variables for the QoL analysis population are summarized in Table 1***.*** Patients were heavily pretreated, with a mean number of prior treatments of 7.9 (SD 3.1, range 3 to 18), and a mean duration of myeloma of 7.6 years (SD 3.4, range 1.2 to 18.6). Excluded patients were similar to the HRQoL analysis population with respect to mean age (65.0 vs 63.7 years), mean number of previous regimens (7.2 vs 7.9), and mean time from initial diagnosis (6.6 vs 7.6 years). Minor differences were noted with respect to sex (62.5 vs 50% male), proportion of patients with high-risk cytogenetics (59.5 vs 50%), and R-ISS risk score stage II (69.0 vs 61.2%). Given the lack of substantial differences in prognostic factors between the HRQoL analysis population and the excluded patients, the impact of excluding these patients is unlikely to affect the results of the analysis.

**Table 1:** Patient baseline characteristics

Patient baseline characteristics	
N (%) unless otherwise indicated	QoL analysis population (*N* = 80)
Male	50 (62.5)
Mean age (SD), range	63.7 (9.4), 40.4 to 85.9
Race	
White	57 (71)
Black	8 (10)
Other	11 (14)
Missing	4 (5)
ECOG performance status	
0	25 (31)
1	45 (56)
2	7 (9)
Missing	3 (4)
R-ISS	
1	16 (20)
2	49 (61)
3	14 (18)
Missing	1 (1)
High-risk cytogenetic abnormalities (any of del(17p)/p53, t(14;16), t(4;14), or 1q21)	40 (50)
Mean number of previous regimens (SD), range	7.9 (3.1), 3 to 18
Mean years since diagnosis (SD), range	7.6 (3.4), 1.2 to 18.6

*QoL* quality of life, *ECOG* Eastern Cooperative Oncology Group, *R-ISS* Revised International Staging System, *SD* standard deviation

Results from the mixed-effects regression analysis for the FACT-MM total score, FACT-G, FACT-MM TOI, and the MM domain are shown in Table 2. Reported coefficients represent the mean change from baseline as estimated by the mixed effects model where a negative value indicates a relative decline from baseline, and a positive value indicates an improvement from baseline. The number of patients who remained in the study declined with each successive cycle, reflecting the highly advanced nature of disease and the proportion of patients who remained well-enough to continue treatment. Most patients showed a monotonic pattern of missingness. Disregarding the end of treatment, only two patients (2.5%) showed intermittent missingness. Scores for the FACT-MM, FACT-G and FACT-MM TOI decreased from baseline at each cycle and at the end of treatment, with significant decreases observed in early cycles of treatment as well as end of treatment. The MM domain score did not change significantly at any cycle or at end of treatment.

**Table 2:** Change in HRQoL as evaluated by a mixed-effects regression model

		Change in FACT-MM total score^a^		Change in FACT-G score^a^		Change in FACT-MM TOI total score^b^		Change in MM domain score^c^	
	Max N	Coefficient (95% CI)	***p***-value	Coefficient (95% CI)	***p***-value	Coefficient (95% CI)	***p***-value	Coefficient (95% CI)	***p***-value
Cycle 2	71^d^	−3.94 (−8.03 to 0.16)	0.059	−3.87 (−6.66 to −1.08)	0.007	−4.86 (−8.44 to −1.29)	0.008	−0.10 (−2.11 to 1.92)	0.926
Cycle 3	42	−5.17 (−10.10 to −0.25)	0.040	−5.69 (−9.05 to −2.33)	0.001	−4.92 (−9.21 to −0.64)	0.024	0.61 (−1.81 to 3.03)	0.619
Cycle 4	25	−5.47 (−11.38 to 0.45)	0.070	−4.00 (−8.04 to 0.04)	0.052	−5.65 (−10.79 to −0.52)	0.031	−1.35 (−4.28 to 1.58)	0.367
Cycle 5	13	−3.97 (−11.58 to 3.65)	0.307	−3.62 (−8.82 to 1.59)	0.173	−4.60 (−11.22 to 2.03)	0.174	0.21 (−3.43 to 3.85)	0.911
Cycle 6	8^e^	−3.54 (−13.59 to 6.51)	0.490	−4.62 (−11.49 to 2.25)	0.187	−3.35 (−12.10 to 5.41)	0.453	2.57 (−1.95 to 7.09)	0.265
End of treatment	39^f^	−10.45 (−16.19 to −4.70)	< 0.0001	−8.14 (−11.68 to −4.60)	< 0.001	−9.39 (−14.22 to −4.57)	< 0.001	−1.80 (−4.93 to 1.34)	0.261

*AIC* Akaike’s Information Criterion, *BIC* Bayesian Information Criterion, *FACT-G* Functional Assessment of Cancer Therapy – General, *FACT-MM* Functional Assessment of Cancer Therapy – Multiple Myeloma, *MM* multiple myeloma, *TOI* Trial Outcomes Index, *CI* confidence interval

^a^ Adjusted for baseline score, race, sex, and years since diagnosis

^b^ Adjusted for baseline score, race, sex, and number of prior regimens

^c^ Adjusted for baseline score, race, sex, ECOG performance score, and number of prior regimens

^d^*n* = 70 for FACT-MM total score and FACT-G

^e^*n* = 7 for FACT-MM total score, FACT-G, and FACT-TOI

^f^*n* = 38 for FACT-MM total score, FACT-G, and FACT-TOI

Next, minimal clinically meaningful changes based on the FACT-MM were evaluated using two anchor-based approaches; the first based on a 10% difference in scale range, and the second based on mean baseline differences between ECOG groups, adjusted for baseline characteristics of age and race. The minimal clinically important difference thresholds calculated for the two approaches are shown in Table 3. These thresholds can be interpreted as the smallest clinically meaningful change for a particular domain. For example, a decrease of the FACT-MM total score by 13.5 points from baseline would represent a clinically meaningful decline as defined by the ECOG-based anchor.

**Table 3:** Minimal clinically important difference thresholds for two anchor-based approaches

	10% difference in score range	Mean baseline difference between ECOG performance scores 0 vs 1 to 2
**Total FACT-MM**	16.4	13.5
**FACT-G**	10.8	7.8
**FACT-TOI**	8.4	11.0
**MM Domain**	5.6	5.8

*ECOG* Eastern Cooperative Oncology Group, *FACT-G* Functional Assessment of Cancer Therapy – General, *FACT-MM* Functional Assessment of Cancer Therapy – Multiple Myeloma, *MM* multiple myeloma, *TOI* Trial Outcomes Index

The number and proportion of patients who improved, experienced no change, or declined in HRQoL based on the minimal clinically important difference anchored by ECOG groups is shown in Table 4. The results of the analysis based on minimal clinically important difference anchored by a 10% difference in scale range is shown in the Supplementary Information. The combined proportions of patients who experienced no change in HRQoL or improvements compared to baseline through cycle 6 based on the FACT-MM total score, FACT-G, FACT-MM TOI, and the MM domain were generally greater than the proportions who experienced declines. Results of the analyses, according to the two minimal clinically important difference definitions, were consistent.

**Table 4:** Patients with improvement, no change, or decline in HRQoL based on minimal clinically important differences defined by an ECOG-based anchor

		Total FACT-MM; n (%) ^a^			FACT-G; n (%) ^b^			FACT-MM TOI; n (%) ^c^			MM Domain; n (%) ^d^		
	Max N	Improvement	No change	Decline	Improvement	No change	Decline	Improvement	No change	Decline	Improvement	No change	Decline
Cycle 2	71 ^e^	9 (12.9)	42 (60.0)	19 (27.1)	9 (12.9)	38 (54.3)	23 (32.9)	10 (14.1)	36 (50.7)	25 (35.2)	14 (19.7)	39 (54.9)	18 (25.4)
Cycle 3	42	4 (9.5)	25 (59.5)	13 (31.0)	4 (9.5)	17 (40.5)	21 (50.0)	6 (14.3)	22 (52.4)	14 (33.3)	9 (21.4)	25 (59.5)	8 (19.1)
Cycle 4	25	2 (8.0)	14 (56.0)	9 (36.0)	4 (16.0)	10 (40.0)	11 (44.0)	1 (4.0)	14 (56.0)	10 (40.0)	1 (4.0)	17 (68.0)	7 (28.0)
Cycle 5	13	4 (30.8)	3 (23.1)	6 (46.2)	4 (30.8)	2 (15.4)	7 (53.9)	3 (23.1)	2 (15.4)	8 (61.5)	1 (7.7)	7 (53.9)	5 (38.5)
Cycle 6	8 ^f^	2 (28.6)	2 (28.6)	3 (42.9)	2 (28.6)	2 (28.6)	3 (42.9)	2 (28.6)	2 (28.6)	3 (42.9)	2 (25.0)	5 (62.5)	1 (12.5)
End of treatment	38	3 (7.9)	17 (44.7)	18 (47.4)	3 (7.9)	11 (29.0)	24 (63.2)	3 (7.9)	14 (36.8)	21 (55.3)	10 (25.6)	13 (33.3)	16 (41.0)

*FACT-G* Functional Assessment of Cancer Therapy – General, *FACT-MM* Functional Assessment of Cancer Therapy – Multiple Myeloma, *MM* multiple myeloma, *TOI* Trial Outcomes Index

^a^ Minimal clinically important difference based on mean difference (13.5 points) in baseline FACT-MM total score between patients with ECOG 0 vs ECOG 1 to 2

^b^ Minimal clinically important difference based on mean difference (7.8 points) in baseline FACT-G score between patients with ECOG 0 vs ECOG 1 to 2

^c^ Minimal clinically important difference based on mean difference (11.0 points) in baseline FACT-MM TOI score between patients with ECOG 0 vs ECOG 1 to 2

^d^ Minimal clinically important difference based on mean difference (5.8) in baseline MM domain score between patients with ECOG 0 vs ECOG 1 to 2

^e^ For FACT-MM and FACT-G, *n* = 70

^f^ For FACT-MM and FACT-G, *n* = 7

Post-hoc analysis evaluated trends in HRQoL change from baseline to end of treatment between treatment responders and non-responders using a mixed-effects model. It should be noted that in the QoL dataset, there were 21 responders (26%; *n* = 21/80), suggesting that responders were no more likely than non-responders to complete the FACT-MM assessment [16]. Results of the difference analysis are summarized in Table 5. With the exception of the MM domain, the negative values for mean change indicate that HRQoL was decreasing for responders and non-responders. The FACT-MM, FACT-G, FACT-MM TOI, and the MM domain scores of non-responders showed a greater decrease from baseline to end of treatment. In contrast, responders had no change as evidenced by positive values of the mean difference. This observed mean difference was significant for the FACT-G (*p* = 0.043), but not other scales.

**Table 5:** Difference analysis results

	Responders (*N* = 21)	Non-responders (*N* = 59)	Mean difference^a^
	Mean change from baseline (95% CI)	Mean change from baseline (95% CI)	(95% CI) *p*-value
**Total FACT-MM**	−1.72 (−13.63 to 10.20)	−12.89 (−19.18 to −6.60)	11.17 (−2.30 to 24.65) *p* = 0.104
**FACT-G**	−3.05 (−8.90 to 2.79)	−10.28 (−14.13 to −6.42)	7.22 (0.22 to 14.22) *p* = 0.043
**FACT-MM TOI**	−4.33 (−14.11 to 5.44)	−10.60 (−15.84 to −5.35)	6.26 (−4.82 to 17.35) *p* = 0.268
**MM Domain**	2.57 (−3.05 to 8.19)	−2.16 (−5.60 to 1.27)	4.73 (−1.85 to 11.32) *p* = 0.159

*FACT-G* Functional Assessment of Cancer Therapy – General, *FACT-MM* Functional Assessment of Cancer Therapy – Multiple Myeloma, *MM* multiple myeloma, *TOI* Trial Outcomes Index, *CI* confidence interval

^a^ Difference analysis between baseline and end of treatment in responders compared to non-responders as estimated by a mixed effects model

## Discussion

Despite advances made in the treatment of RRMM, the disease remains incurable and patients with RRMM face a significant burden due to symptoms, treatment-associated adverse events, and cumulative toxicities of prior therapies [11–13]. Inclusion of QoL evidence is an important factor in treatment decision-making that aims to balance clinical efficacy of newer therapies with the burden of adverse events, particularly among heavily pretreated patients with advanced disease [13]. Several studies have examined HRQoL in patients with RRMM receiving doublet or triplet therapies, daratumumab, or autologous stem cell transplantation with a variety of disease specific instruments [17, 30–39]. Maintenance in HRQoL was observed in randomized controlled trials (RCTs) with pomalidomide and low-dose dexamethasone [39], panobinostat, bortezomib, and dexamethasone [36], daratumumab [31], pomalidomide, bortezomib, and dexamethasone [38], and carfilzomib and dexamethasone [34] in later lines of therapy (3 L+).

The current analysis examined HRQoL effects in patients with penta-refractory MM, who received selinexor and low dose dexamethasone in the STORM phase 2b trial. FACT-MM, FACT-G, and FACT-MM TOI scores of patients declined significantly from baseline in the early cycles and at the end of treatment, while significant changes were not observed in the MM-specific domain at any point from baseline. Two anchors were utilized to estimate minimal clinically important difference and yielded consistent findings. Anchor-based approaches have been utilized to establish minimal clinically important difference for other FACT instruments [40–44]. No known, validated minimal clinically important difference has been reported for the FACT-MM. The exploratory approach utilizing patient-level data was based on the previously established relationship between QoL outcomes and ECOG PS scores [20]. This is in contrast to distribution-based minimal clinically important difference evaluations, which do not consider clinical reference points and are only statistical by nature. The observed consistency in findings with the previously applied distribution-based approach serves as a validation of the novel anchor-based approach.

A key finding of the analysis is that, generally, the combined proportions of patients who experienced no change in HRQoL or improvements were higher compared to those who experienced declines in the early cycles of treatment with selinexor and dexamethasone according to minimal clinically important differences. In addition, the difference analysis identified that treatment responders had less HRQoL decline than non-responders. An association between HRQoL and response to treatment has been observed in previous RCTs. A significant improvement in HRQoL was observed in patients with partial or complete response with bortezomib in the SUMMIT phase 2 trial, while deterioration in HRQoL was observed among patients who did not respond and had progressive disease based on the European Organisation for Research and Treatment of Cancer Quality of Life Questionnaire C-30 (EORTC QLQ-C30) [45]. In the carfilzomib, lenalidomide, and dexamethasone arm of the phase 3 RCT ASPIRE, patients achieving a partial response or better had significantly higher HRQoL over 18 cycles of treatment compared with patients who did not respond to treatment, according to the Global Health Status scale of the EORTC QLQ-C30 [37]. The limited impact on HRQoL, particularly among treatment responders, suggests a favorable benefit-risk profile of selinexor, given its demonstrated efficacy and tolerability among patients with penta-refractory MM, and considering the unmet therapeutic need in this patient population.

### Limitations

An important limitation of the analysis is the single-arm study design of the STORM phase 2b trial, which did not enable comparison of HRQoL outcomes with a comparator arm who received conventional medical management. As a result, treatment-associated changes in HRQoL cannot be directly extracted from the analysis. HRQoL was a secondary endpoint, and all analyses and results should be considered as explorative. The analysis aimed to examine change in HRQoL during, and at end of treatment with selinexor and low dose dexamethasone. The analysis did not investigate treatment-associated adverse events that may have been associated with changes in HRQoL due to the small sample size of the HRQoL dataset and high attrition rates seen in STORM; considering these factors, the study would be underpowered to detect significant differences.

Another limitation of the analysis was the small sample size of patients with post-baseline HRQoL data. The sample size decreased over time, particularly in later cycles (i.e., cycle 7 or greater) that were omitted from the analysis due to sparse numbers. Observed differences in the composition of patients with minimal clinically important differences may be attributed to attrition, particularly among non-responders. Compliance rates were good in earlier cycles up to cycle 5, with ≥65% of patients on treatment completing the FACT-MM, however a decline to 54% was observed in cycle 6. Combined with the small sample size, statistical power to detect changes in scores may be further reduced for each covariate added to the model.

The mixed-effects models assumed an MAR pattern of data, which presumes that all characteristics associated with missingness were adjusted for in the model. The MAR assumption was tested by examining patterns of missingness in the trial. Because different groups of patients were observed at each cycle, baseline FACT-MM scores also varied across each cycle. The moving baseline values and the MAR assumption should be taken into consideration in the interpretation of observed HRQoL changes.

Lastly, treatment responders were not randomized. As a result, significant differences between responders and non-responders could be present between HRQoL at baseline or for other baseline characteristics. A difference analysis was performed since subgroup analysis according to response was not well powered to perform statistical testing. It should be noted that the difference analysis is a quasi-experimental approach, which has been utilized in epidemiologic studies, but has not been commonly used for HRQoL analyses [46].

## Conclusions

The current analysis examined patient-reported HRQoL in the STORM mITT population using the FACT-MM. Minimal clinically important difference analyses demonstrated that most patients did not experience HRQoL decline during early cycles of treatment with selinexor and low dose dexamethasone. Exploratory minimal clinically important differences, defined as 10% of the instrument range or as an ECOG-based anchor, yielded consistent results. Treatment responders were found to experience less decline in HRQoL from baseline to end of treatment than non-responders, which was significant only for the FACT-G. Important limitations of the analysis were the single-arm study design and the limited sample size. Overall findings complement the demonstrated efficacy and tolerability of selinexor with low dose dexamethasone in patients with penta-refractory MM.

## Supplementary Information

**Additional file 1 Table 1***.* Patients with improvement, no change, or decline in HRQoL based on minimal clinically important differences defined by ≥ 10% of the instrument range. **Table 1** describes the number and proportion of patients with improvement, no change, or decline in HRQoL based on the minimal clinically important difference threshold defined as ≥10% of the instrument range. Data are shown for the FACT-MM, FACT-G, FACT-MM TOI, and the MM domain at treatment cycle 2–6 and end of treatment.

**Additional file 2.** Provides a list of IECs and IRBs for the STORM trial.

## Data Availability

The datasets analyzed in this work may be available from the corresponding author on reasonable request and permission of Karyopharm Therapeutics Inc.

## Abbreviations

AIC: Akaike’s Information Criterion
BIC: Bayesian Information Criterion
CI: Confidence interval
ECOG: Eastern Cooperative Oncology Group
EORTC QLQ-C30: European Organisation for Research and Treatment of Cancer Quality of Life Questionnaire C-30
FACT-G: Functional Assessment of Cancer Therapy – General
FACT-MM: Functional Assessment of Cancer Therapy – Multiple Myeloma
FACT-TOI: Functional Assessment of Cancer Therapy – Trial Outcomes Index
HRQoL: Health-related quality of life
MAR: Missing at random
mITT: Modified intent-to-treat
MM: Multiple myeloma
QoL: Quality of life
RCT: Randomized controlled trial
R-ISS: Revised International Staging System
RRMM: Relapsed/refractory multiple myeloma
SD: Standard deviation
STORM: Selinexor Treatment of Refractory Myeloma
