# Supplementary material for: Quality of life analyses in patients with multiple myeloma: results from the Selinexor (KPT-330) Treatment of Refractory Myeloma (STORM) phase 2b study
Source: BMC Cancer. 2021 Sep 6;21:993. doi: 10.1186/s12885-021-08453-9 (PMC8419947; doi:10.1186/s12885-021-08453-9)
Supplement: Supplementary file 1 — Additional file 1 Table 1. Patients with improvement, no change, or decline in HRQoL based on minimal clinically important differences defined by ≥ 10% of the instrument range. Table 1 describes the number and proportion of patients with improvement, no change, or decline in HRQoL based on the minimal clinically important difference threshold defined as ≥10% of the instrument range. Data are shown for the FACT-MM, FACT-G, FACT-MM TOI, and the MM domain at treatment cycle 2–6 and end of treatment. [file 12885_2021_8453_MOESM1_ESM.docx]

# Appendix

**Table 1:** Patients with improvement, no change, or decline in HRQoL based on minimal clinically important differences defined by ≥10% of the instrument range

|  |  | Total FACT-MM; n (%) ^a^ | |  | FACT-G; n (%) ^b^ | |  | FACT-MM TOI; n (%) ^c^ | |  | MM domain; n (%) ^d^ | |  |
| --- | --- | --- | --- | --- | --- | --- | --- | --- | --- | --- | --- | --- | --- |
|  | **Max N** | **Improvement** | **No change** | **Decline** | **Improvement** | **No change** | **Decline** | **Improvement** | **No change** | **Decline** | **Improvement** | **No change** | **Decline** |
| Cycle 2 | 71 ^e^ | 8 (11.4) | 45 (64.3) | 17 (24.3) | 8 (11.4) | 44 (62.9) | 18 (25.7) | 11 (15.5) | 34 (47.9) | 26 (36.6) | 14 (19.7) | 39 (54.9) | 18 (25.4) |
| Cycle 3 | 42 | 4 (9.5) | 27 (64.3) | 11 (26.2) | 4 (9.5) | 23 (54.8) | 15 (35.7) | 7 (16.7) | 20 (47.6) | 15 (35.7) | 9 (21.4) | 25 (59.5) | 8 (19.1) |
| Cycle 4 | 25 | 2 (8.0) | 16 (64.0) | 7 (32.0) | 2 (8.0) | 14 (56.0) | 9 (36.0) | 1 (4.0) | 12 (48.0) | 12 (48.0) | 1 (4.0) | 17 (68.0) | 7 (28.0) |
| Cycle 5 | 13 | 3 (23.1) | 4 (30.8) | 6 (46.2) | 3 (23.1) | 3 (23.1) | 7 (53.9) | 4 (30.8) | 1 (7.7) | 8 (61.5) | 1 (7.7) | 7 (53.9) | 5 (38.5) |
| Cycle 6 | 8 ^f^ | 1 (14.3) | 3 (42.9) | 3 (42.9) | 1 (14.3) | 3 (42.9) | 3 (42.9) | 3 (42.9) | 1 (14.3) | 3 (42.9) | 3 (37.5) | 4 (50.0) | 1 (12.5) |
| End of treatment | 38 | 3 (7.9) | 19 (50.0) | 16 (42.0) | 1 (2.6) | 19 (50.0) | 18 (47.4) | 4 (10.5) | 11 (29.0) | 23 (60.5) | 10 (25.6) | 13 (33.3) | 16 (41.0) |

FACT-G: Functional Assessment of Cancer Therapy – General; FACT-MM: Functional Assessment of Cancer Therapy – Multiple Myeloma; MM: multiple myeloma; TOI: Trial Outcomes Index

^a^ Minimal clinically important difference based on ≥10% difference in FACT-MM range (16.4 points)

^b^ Minimal clinically important difference based on ≥10% difference in FACT-G range (10.8 points)

^c^ Minimal clinically important difference based on ≥10% difference in FACT-MM TOI range (8.4 points)

^d^ Minimal clinically important difference based on ≥10% difference in MM domain range (5.6 points)

^e^ For FACT-MM and FACT-G, n=70

^f^ For FACT-MM and FACT-G, n=7
