# Supplementary material for: Quality of life analyses in patients with multiple myeloma: results from the Selinexor (KPT-330) Treatment of Refractory Myeloma (STORM) phase 2b study
Source: BMC Cancer. 2021 Sep 6;21:993. doi: 10.1186/s12885-021-08453-9 (PMC8419947; doi:10.1186/s12885-021-08453-9)
Supplement: Supplementary file 2 — Additional file 2. Provides a list of IECs and IRBs for the STORM trial. [file 12885_2021_8453_MOESM2_ESM.pdf]

16.1.3 LIST OF IECS OR IRBS AND REPRESENTATIVE WRITTEN INFORMATION FOR PATIENT AND SAMPLE CONSENT FORMS

List of Ethics Committees

Site No.	Investigator Name	Name / Address of Ethics Committee	Name / Address of Central Ethics Committee
0001	Rachid Baz, MD	Liberty IRB<br>1450 S. Woodland Blvd<br>Deland, FL 32720<br><br>Chesapeake IRB / Advarra<br>6940 Columbia Gateway Drive, Suite 110<br>Columbia, MD 21046	
0002	William I. Bensinger, MD	Western Institutional Review Board<br>1019 39th Avenue SE<br>Suite 120<br>Puyallup, WA 98374-2115	
0003	Nashat Gabrail, MD	Western Institutional Review Board<br>1019 39th Avenue SE<br>Suite 120<br>Puyallup, WA 98374-2115	
0004	Andrzej Jakubowiak, MD, PhD	University of Chicago IRB<br>McGiffert House, 2nd Floor<br>5751 Woodlawn Ave<br>Chicago, IL 60637<br><br>AURA<br>University Research Administration<br>6054 S. Drexel Ave Suite 300<br>Chicago, Illinois 60637<br><br>AURA<br>University Research Administration<br>5841 S. Maryland Avenue<br>Chicago, IL 60637	
0005	Suzanne Lentzsch, MD, PhD	Columbia University Medical Center<br>154 Haven Avenue, Floor 1<br>New York, NY 10032	

Site No.	Investigator Name	Name / Address of Ethics Committee	Name / Address of Central Ethics Committee
0006	Ajay Nooka, MD, MPH, FACP	Emory IRB<br>1599 Cliffton Road NE<br>Atlanta GA 30322	
0007	Amithaba Mazumder, MD / David Kaminetzky, MD	NYU School of Medicine IRB<br>1 Park Avenue, 6th Floor<br>New York, NY 10016	
0008	Kevin Thomas McDonagh, MD / Robert Frank Cornell, MD	Vanderbilt University IRB<br>1313 21st Avenue South<br>Nashville, TN 37232-4315	
0010	Gary Schiller, MD	University of California Los Angeles<br>11000 Kinross Avenue, Suite 211<br>Los Angeles, CA 90095-1694	
0012	Joshua Richter, MD /<br>Noa Biran, MD	Western Institutional Review Board<br>1019 39th Avenue SE Suite 120<br>Puyallup, WA 98374-2115	
0013	Keith Stewart, MD, PhD	Mayo Clinic IRB<br>220 First Street SW<br>Rochester, MN 55905	
0015	Jeff Allan Zonder, MD /<br>Divaya Bhutani, MD	Wayne State University (WSU) -<br>87 E Canfield. Second Floor,<br>Detroit MI 48201	
0016	Keith Stewart, MD PhD<br><br>David Dingli, MD, PhD	Mayo Clinic IRB<br>220 First Street SW<br>Rochester, MN 55905	
0018	Andrew Yee, MD	Office for Human Research Studies<br>Dana Farber/Harvard Cancer Institute<br>450 Brookline Avenue (OS-229)<br>Boston, MA 02215	

Site No.	Investigator Name	Name / Address of Ethics Committee	Name / Address of Central Ethics Committee
0019	Rafat M. Abonour, MD<br>Attaya Suvannasankha, MD	Western Institutional Review Board<br>1019 39th Avenue SE<br>Suite 120<br>Puyallup, WA 98374-2115	
0020	Ray Comenzo, MD	Western Institutional Review Board<br>1019 39th Avenue SE<br>Suite 120<br>Puyallup, WA 98374-2115	
0021	Sundar Jagannath, MD	BRANY<br>1981 Marcus Avenue, Suite 210<br>Lake Success, NY 11042	
0022	Ravi Vij, MD, MBA	Washington University School of Medicine<br>Human Research Protection Office<br>660 S. Euclid Ave. – Box 8089<br>St. Louis, MO 63110	
0026	Dan Vogl, MD, M.S.C.E.	University of Pennsylvania<br>Office of Regulatory Affairs<br>3624 Market St.,<br>Suite 301 S<br>Philadelphia, PA 19104-6006<br><br>University of Pennsylvania<br>Office of Regulatory Affairs<br>3800 Spruce Street – First Floor<br>Suite 151<br>Philadelphia, PA 19104	
0027	Michael Rosenzweig, MD	Western Institutional Review Board<br>1019 39th Avenue SE<br>Suite 120<br>Puyallup, WA 98374-2115	
0028	Melhelm Solh, MD	Western Institutional Review Board<br>1019 39th Avenue SE<br>Suite 120<br>Puyallup, WA 98374-2115	

Site No.	Investigator Name	Name / Address of Ethics Committee	Name / Address of Central Ethics Committee
0031	James J. Hoffman, MD	University of Miami<br>Human Subject Research Office (M809)<br>1400 NW 10th Avenue, Suite 1200A<br>Miami, FL 33136	
0032	Luciano Costa, MD, PhD	Western Institutional Review Board<br>1019 39th Avenue SE<br>Suite 120<br>Puyallup, WA 98374-2115	
0033	Carol Ann Huff, MD	Office of Human Subjects Research<br>Institutional Review Boards<br>1620 McElderry Street, Reed Hall, Suite B-130<br>Baltimore, Maryland 21205-1911	
0034	Craig Cole, MD	University of <Michigan<br>Medical School Institutional Review Board (IRBMED)<br>2800 Plymouth Road<br>Building 520<br>Ann Arbor, MI 48109-2800	
0035	Terri Lynn Parker, MD	BRANY<br>1981 Marcus Avenue<br>Suite 210<br>Lake Success, NY 11042	
0036	Jason Suh, MD	BRANY<br>1981 Marcus Avenue<br>Suite 210<br>Lake Success, NY 11042	
0037	Andrew Yee, MD<br><br>Robert Schlossman, MD<br>Paul Richardson, MBBS, MA	Office for Human Research Studies<br>Dana Farber/Harvard Cancer Institute<br>450 Brookline Avenue (OS-229)<br>Boston, MA 02215	

Site No.	Investigator Name	Name / Address of Ethics Committee	Name / Address of Central Ethics Committee
0038	Moshe Levy, MD	Baylor Research Institute IRB<br>3310 Live Oak, Suite 501 -<br>Dallas, TX 75204	
0039	Sascha Alexander Tuchman, MD, MHS	University of North Carolina at Chapel Hill<br>Office of Human Research Ethics<br>720 Martin Luther King, Jr. Blvd.<br>Bldg. 385, 2nd Floor<br>CB #7097<br>Chapel Hill, NC 27599-7097	
0040	Hemant S. Murthy, MD	Western Institutional Review Board<br>1019 39th Avenue SE<br>Suite 120<br>Puyallup, WA 98374-2115	
0045	Donald MacFarlane, MD, PhD	University of Iowa<br>Human Subjects Office /<br>Institutional Review Board<br>Hardin Library for the Health Sciences – Office 105<br>600 Newton Road<br>Iowa City, Iowa 52242-1098	
0046	Jennifer Fu Carney, MD	Kaiser Permanente Hawaii IRB<br>Center for Health Research-Hawaii<br>711 Kapiolani Blvd – Suite 211<br>Honolulu, HI 96813	
0048	Abdul Monsoor, MD	Kaiser Permanente Northwest<br>Research Subjects Protection Office<br>3800 North Interstate Avenue<br>Portland, OR 97227	
0049	Don Stevens, MD	Western Institutional Review Board<br>1019 39th Avenue SE<br>Suite 120<br>Puyallup, WA 98374-2115	
0051	Pallawi Torka, MD	Roswell Park Cancer Institute IRB<br>Elm & Carlton Streets<br>Buffalo, NY 14263	

Site No.	Investigator Name	Name / Address of Ethics Committee	Name / Address of Central Ethics Committee
2201	Maria Krauth	Ethikkommission der Medizinischen Universität Wien<br>Borschkegasse 8b/E06<br>1090 Wien (Austria)	Ethics Committee of Vienna Medical University<br>[Ethikkommission der Medizinischen Universität Wien]<br>Borschkegasse 8b/6<br>1090 Vienna, Austria
2202	Richard Greil	Ethikkommission für das Bundesland Salzburg<br>Amt der Salzburger Landesregierung<br>Postfach 527<br>5010 Salzburg (Austria)	Ethics Committee of Vienna Medical University<br>[Ethikkommission der Medizinischen Universität Wien]<br>Borschkegasse 8b/6<br>1090 Vienna, Austria
2203	Klaus Podar	Ethikkommission für das Land Niederösterreich, Am Sitz des Amtes NÖ Landesregierung<br>Abteilung Gesundheitswesen<br>Landhausplatz 1, Haus 15B<br>3109 St. Pölten (Austria)	Ethics Committee of Vienna Medical University<br>[Ethikkommission der Medizinischen Universität Wien]<br>Borschkegasse 8b/6<br>1090 Vienna, Austria
0401	Michel Delforge	Medical Ethics Committee of the Leuven University Hospital; Herestraat 49 3000 Leuven	Ethics Committee /UZ Leuven<br>Herestraat 49, B 3000 Leuven<br>Belgium
0402	Nathalie Meuleman	Comité d'éthique<br>INSTITUT JULES BORDET<br>1000 Brussels	Ethics Committee /UZ Leuven<br>Herestraat 49, B 3000 Leuven<br>Belgium
0403	Philip Vlummens	Ethics Committee of the Ghent University Hospital	Ethics Committee /UZ Leuven<br>Herestraat 49, B 3000 Leuven<br>Belgium
0404	Ka Lung Wu	Commissie voor Medische Ethiek ZNA, p/a ZNA Koningin Paola Kinderziekenhuis, P4, Route 34, Lindendreef 1, 2020 Antwerpen	Ethics Committee /UZ Leuven<br>Herestraat 49, B 3000 Leuven<br>Belgium
0405	Chantal Doyen	Comité d'éthique<br>C.H.U. – UCL MONT-GODINNE - DINANT<br>5530 Mont-Godinne	Ethics Committee /UZ Leuven<br>Herestraat 49, B 3000 Leuven<br>Belgium
0406	Jan Van Droogenbroeck	AZ Sint-Jan Brugge-Oostende AV<br>COMMISSIE VOOR ETHIEK<br>Ruddershove 10<br>8000 Brugge	Ethics Committee /UZ Leuven<br>Herestraat 49, B 3000 Leuven<br>Belgium

Site No.	Investigator Name	Name / Address of Ethics Committee	Name / Address of Central Ethics Committee
0407	Marie-Christine Vekemans	Comité d'ethique Saint Luc<br>Promenade de l'Alma 51 bte B1.43.03 (au-dessus du métro Alma)<br>1200 Bruxelles	Ethics Committee /UZ Leuven<br>Herestraat 49, B 3000 Leuven<br>Belgium
0801	Facon	Not applicable	Comite de Protection de Personnes Ouest VI (CPP)<br>CPP OUEST 6<br>CHU Cavale Blanche 29609<br>BREST CEDEX
0802	Frenzel	Not applicable	Comite de Protection de Personnes Ouest VI (CPP)<br>CPP OUEST 6<br>CHU Cavale Blanche 29609<br>BREST CEDEX
0803	Karlin	Not applicable	Comite de Protection de Personnes Ouest VI (CPP)<br>CPP OUEST 6<br>CHU Cavale Blanche 29609<br>BREST CEDEX
0804	Moreau	Not applicable	Comite de Protection de Personnes Ouest VI (CPP)<br>CPP OUEST 6<br>CHU Cavale Blanche 29609<br>BREST CEDEX
0805	Mohty	Not applicable	Comite de Protection de Personnes Ouest VI (CPP)<br>CPP OUEST 6<br>CHU Cavale Blanche 29609<br>BREST CEDEX
0806	Choquet	Not applicable	Comite de Protection de Personnes Ouest VI (CPP)<br>CPP OUEST 6<br>CHU Cavale Blanche 29609<br>BREST CEDEX
0807	Perrot	Not applicable	Comite de Protection de Personnes Ouest VI (CPP)<br>CPP OUEST 6<br>CHU Cavale Blanche 29609<br>BREST CEDEX

Site No.	Investigator Name	Name / Address of Ethics Committee	Name / Address of Central Ethics Committee
0201	Monika Engelhardt	Ethikkommission der Albert-Ludwigs-Universität Freiburg<br>Engelbergerstr. 21<br>79106 Freiburg	Ethik Kommission/ Albert-Ludwigs Universität Freiburg<br>Engelberger Strasse 21, 79106 Freiburg
0202	Nicole Adrian	Ethikkommission bei der Ärztekammer des Saarlandes<br>Faktoreistr. 4<br>666111 Saarbrücken	Ethik Kommission/ Albert-Ludwigs Universität Freiburg<br>Engelberger Strasse 21, 79106 Freiburg
0203	Stefan Knop	Ethikkommission an der Medizinischen Fakultät der Universität Würzburg<br>Versbacher Strasse 9<br>97078 Würzburg	Ethik Kommission/ Albert-Ludwigs Universität Freiburg<br>Engelberger Strasse 21, 79106 Freiburg
0204	Markus Munder	Ethikkommission bei der Landesärztekammer Rheinland-Pfalz<br>Deutschhausplatz 3<br>55116 Mainz	Ethik Kommission/ Albert-Ludwigs Universität Freiburg<br>Engelberger Strasse 21, 79106 Freiburg
0205	Marc Steffen Raab	Ethikkommission I der Medizinischen Fakultät Heidelberg<br>Alte Glockengießerei 11/1<br>69115 Heidelberg	Ethik Kommission/ Albert-Ludwigs Universität Freiburg<br>Engelberger Strasse 21, 79106 Freiburg
0206	Katja Wiesel	Ethikkommission an der Medizinischen Fakultät der Eberhard-Karls-Universität und am Universitätsklinikum Tübingen<br>Gartenstrasse 47<br>72074 Tübingen	Ethik Kommission/ Albert-Ludwigs Universität Freiburg<br>Engelberger Strasse 21, 79106 Freiburg
0207	Thomas Illmer	Ethikkommission der Sächsischen Landesärztekammer<br>Schützenhöhe 16<br>01099 Dresden	Ethik Kommission/ Albert-Ludwigs Universität Freiburg<br>Engelberger Strasse 21, 79106 Freiburg
2401	Dimopoulos	Scientific Committee of "Alexandra" General Hospital of Athens<br>80 Vassilissis Sofias Avenue<br>115 28 Athens<br>GREECE	HELLENIC REPUBLIC MINISTRY OF HEALTH NATIONAL ETHICS COMMITTEE<br>284 Mesogeion Ave., 155 62 Cholargos
